# Quality‐of‐life, mental health, and perspective on TKI dose reduction as a prelude to discontinuation in chronic phase chronic myeloid leukemia

**DOI:** 10.1002/cam4.6296

**Published:** 2023-07-06

**Authors:** Yilin Chen, Na Xu, Yunfan Yang, Zhenfang Liu, Mengxing Xue, Li Meng, Qun He, Chunyan Chen, Qingshu Zeng, Huanling Zhu, Xin Du, Jing Zou, Wenjun He, Jingming Guo, Suning Chen, Guolin Yuan, Hui Wu, Mei Hong, Fanjun Cheng, Bingcheng Liu, Yanli Zhang, Weiming Li

**Affiliations:** ^1^ Department of Hematology, Union Hospital, Tongji Medical College Huazhong University of Science and Technology Wuhan China; ^2^ Department of Hematology, Nanfang Hospital Southern Medical University Guangzhou China; ^3^ Department of Hematology, West China Hospital Sichuan University Chengdu China; ^4^ Department of Hematology The First Affiliated Hospital of Guangxi Medical University Nanning China; ^5^ National Clinical Research Center for Hematologic Diseases, Jiangsu Institute of Hematology The First Affiliated Hospital of Soochow University Suzhou China; ^6^ Institute of Blood and Marrow Transplantation, Collaborative Innovation Center of Hematology Soochow University Suzhou China; ^7^ Department of Hematology, Tongji Hospital, Tongji Medical College Huazhong University of Science and Technology Wuhan China; ^8^ Department of Hematology Xiangya Hospital of Central South University Changsha China; ^9^ Department of Hematology, Qilu Hospital, Cheeloo College of Medicine Shandong University Jinan China; ^10^ Department of Hematology First Affiliated Hospital of Anhui Medical University Hefei China; ^11^ Department of Hematology and Shenzhen Bone Marrow Transplantation Public Service Platform, Shenzhen Second People's Hospital The First Affiliated Hospital of Shenzhen University Shenzhen China; ^12^ Department of Hematology First Clinical Medical College of China Three Gorges University, Yichang Central People's Hospital Yichang China; ^13^ Department of Hematology Xiangyang Central Hospital, The Affiliated Hospital of Hubei University of Arts and Science Xiangyang China; ^14^ Department of Hematology Hanchuan People's Hospital Hanchuan China; ^15^ State Key Laboratory of Experimental Hematology, National Clinical Research Center for Blood Diseases, Haihe Laboratory of Cell Ecosystem Institute of Hematology and Blood Diseases Hospital, Chinese Academy of Medical Sciences and Peking Union Medical College Tianjin China; ^16^ Department of Hematology The Affiliated Cancer Hospital of Zhengzhou University Zhengzhou China

**Keywords:** chronic myeloid leukemia, mental health, quality‐of‐life, TKI dose reduction, treatment‐free remission

## Abstract

**Background:**

Treatment‐free remission (TFR) has become the main target for chronic myeloid leukemia (CML). Tyrosine kinase inhibitors (TKI) dose optimization is crucial in managing adverse events, and improving adherence in clinical practice. In persons achieving a deep molecular response (DMR), some data suggest TKI dose reduction before discontinuation does not change success rate of achieving TFR, but this is controversial. However, data on quality‐of‐life (QoL) and mental health in CML patients with full‐dose TKI, low‐dose TKI, and TKI discontinuation are limited. Moreover, recent evidence indicating the feasibility of TKI dose reduction and discontinuation after dose reduction, which may change CML patients' perspectives on TKI discontinuation.

**Methods:**

We conducted a cross‐sectional study using online questionnaires to explore the QoL, mental health in patients with diverse TKI dose, and perspective on TKI dose reduction as a prelude to discontinuation.

**Results:**

1450 responses were included in the analysis. 44.3% of respondents reported a moderate‐to‐severe impact of TKI treatment on their QoL. 17% of respondents had moderate‐to‐severe anxiety. 24.4% of respondents had moderate‐to‐severe depression. In 1326 patients who had not discontinued their medication, 1055 (79.6%) patients reported they would try TKI discontinuation because of concerns over side effects of long‐term medication (67.9%), financial burden (68.7%), poor QoL (77.9%), pregnancy needs (11.6%), anxiety and depression while taking TKI (20.8%), inconvenience of TKI treatment (22.2%). 613 of 817 (75.0%) patients on full‐dose TKI therapy indicated they preferred trying a dose reduction before discontinuing TKI therapy after dose reduction compared with 31 (3.8%) preferring no dose reduction before stopping.

**Conclusions:**

TKI dose reduction showed a significant improvement of patients' QoL and mental health, comparable to the effect of TKI discontinuation. Most patients indicated they preferred dose reduction before stopping TKI therapy. In clinical practice, TKI dose reduction can be considered as a bridge from full‐dose treatment to discontinuation.

Our results showed that tyrosine kinase inhibitors (TKI) dose reduction showed a significant improvement of patients' quality‐of‐life and mental health, comparable to the effect of TKI discontinuation. Most patients desire to discontinue TKI in the future. TKI discontinuation after dose reduction is more acceptable compared to discontinuing it directly. In clinical practice, TKI dose reduction can be considered as a bridge from full‐dose treatment to discontinuation. Please do not hesitate to contact me in case further clarification is needed with this submission.

## Graphical abstract

A cross‐sectional study was conducted to explore the quality of life (QoL), mental health in chronic myeloid leukemia (CML) patients with diverse tyrosine kinase inhibitors (TKI) dose, and perspective on TKI dose reduction as a prelude to discontinuation. TKI dose reduction showed a significant improvement of patients' QoL and mental health, comparable to the effect of TKI discontinuation. Most patients preferred dose reduction before discontinuing TKI therapy. In clinical practice, TKI dose reduction can be considered as a bridge from full‐dose TKI treatment to TKI discontinuation.

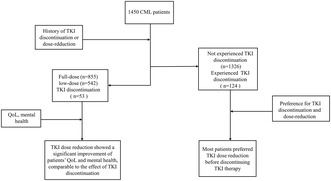

## 1 INTRODUCTION

The emergence of tyrosine kinase inhibitors (TKI) has greatly improved the prognosis of patients in the chronic phase of chronic myeloid leukemia (CML), and most patients can live a near‐normal life expectancy.^1^ However, long‐term TKI use is frequently accompanied by adverse events, impact on quality‐of‐life (QoL), impaired mental health, poor adherence, and high treatment costs.^2^, ^3^, ^4^ In recent years, several clinical trials and real‐world studies have shown that 40%–60% of patients with a sustained, stable deep molecular response (DMR) can successfully discontinue TKI and achieve treatment‐free remission (TFR).^5^, ^6^, ^7^ Currently, patients with more than 5 years of TKI and 3 years of DMR without prior treatment failure should be considered for withdrawal.^8^, ^9^ However, it is worth noting that only a proportion of patients can reach the threshold for TKI discontinuation. In the ENSTnd study, with ≥10 years follow‐up in newly diagnosed CML patients, the estimated cumulative rates of TFR eligibility with nilotinib 300‐mg twice‐daily, nilotinib 400‐mg twice‐daily, and imatinib, respectively, and 10 years were 48.6%, 47.3%, and 29.7%.^10^ Combining other TKI discontinuation data, it is estimated that only 10%–25% of patients could achieve stable TFR.^11^ Similarly, Baccarani et al. recently reported that 70% of patients require continuous TKI for long‐term survival.^12^ In addition, some patients are hesitant to stop TKI therapy for a variety of reasons, frequently for fear of leukemia relapse.^13^

Recently, TKI dose optimization is also being increasingly emphasized as an important part of individualized therapy. Several studies have confirmed that low‐dose TKI can effectively maintain molecular response.^14^ In the non‐randomized DESTINY trial, 2% of patients with MR4.0 and 19% with MMR experienced molecular recurrence after 12 months of half‐dose TKI therapy.^15^ Fassoni et al. developed a mathematical model based on real data from selected patients in the IRIS study and the CML‐IV study, which showed that a reduction in TKI dose of at least 50% did not lead to a deterioration in long‐term treatment outcomes.^16^ Furthermore, long‐term follow‐up of the DESTINY trial presented a 2‐year relapse‐free rate of 72% and 36% for the MR4.0 and MMR groups after TKI discontinuation.^17^ A recent study also reported that dose reduction before discontinuation did not impact the attainment of TFR.^18^ However, data on QoL and mental health in CML patients with full‐dose TKI, low‐dose TKI, TKI discontinuation are limited. In addition, evidence indicating the feasibility of TKI dose reduction and discontinuation after dose reduction may change CML patients' perspectives on TKI discontinuation. Here, we conducted a cross‐sectional study to explore the QoL, mental health in patients with diverse TKI dose and patients' perspective on TKI‐dose reduction as a prelude to discontinuation.

## 2 METHODS

### 2.1 Study design and data collection methods

We designed a cross‐sectional online survey to explore the QoL and mental health in patients with diverse TKI doses, and patients' perceptions of TKI dose reduction and TKI discontinuation. The CROSS guidelines were utilized to report on the findings.^19^ The survey included four sections with 29 items covering patient and treatment characteristics, history of TKI discontinuation and TKI dose reduction, patient's self‐reported events such as QoL, mental health, and preference of TFR and dose reduction and reason. The first dimension included 13 items assessing patient and treatment characteristics, consisting of domains of age, sex, marriage, and educational level, CML‐related data such as diagnosis, therapy, response, and financial burden. The financial burden of treatment was assessed on a scale of 1–4 (1 = no, 4 = severe large). The second dimension included six questions about the history of TKI discontinuation and TKI dose reduction. The third included four questions assessing patients' self‐reported events such as QoL and mental health. The impact of TKI treatment on their QoL was assessed on a scale of 1–5 (1 = no impact; 5 = high impact). Mental health was evaluated by the 9‐item Patient Health Questionnaire (PHQ‐9) and the 7‐item Generalized Anxiety Disorder (GAD‐7).^20^, ^21^ Depressive symptoms were classified according to the total PH9 score as control (0–4), mild (5–9), moderate (10–14), and severe (15–27). Anxiety symptoms were categorized according to the GAD‐7 as follows: controls (0–4), mild (5–9), moderate (10–14), and severe (15–21). The fourth included six questions about treatment goals, attitudes, and reasons for TFR and TKI dose reduction for CML patients. The full survey is included in Supplementary Materials.

### 2.2 Sample characteristics

From February 28, 2022 to April 12, 2022, a cross‐sectional online survey was conducted using the WeChat‐based survey program Wenjuanxing. Patients with a CML diagnosis were eligible to participate. The online questionnaire was distributed through patient WeChat groups in 20 provinces. Data of 1633 respondents with CML from 20 provinces were collected. Questionnaires from subjects <16 years (*n* = 20), duplicates (*n* = 66), incomplete (*n* = 97) were excluded. A total of 1450 respondents were included in the analysis.

### 2.3 Ethical considerations

This study was approved by the Ethics Committee of Union Hospital, Tongji Medical College, Huazhong University of Science and Technology and informed consent was waived by the Institutional Review Board.

### 2.4 Statistical analyses

Descriptive analysis results were presented as median (range) or number (percent). Covariates with *p* < 0.05 in univariate analysis were included in the multivariate binary logistic regression analyses. *p* < 0.01 was considered statistically significant. Analyses were conducted with SPSS Version 22.0 software.

## 3 RESULTS

### 3.1 Patient and treatment characteristics

From February 28 to April 12, 2022, 1450 responses were included in the analysis. 54.8% (795) of respondents were male, and the median age was 44 (with a range of 16–83). 1356 (93.5%) were in the chronic phase at diagnosis. Notably, the proportion of senior middle school and higher education in our study is actually higher than the general population. This is also the bias of online questionnaires that higher educated and younger aged patients responded more than their counterparts.

The median TKI therapy duration was 50 months (range, 1–263 months). 89 (6.1%) respondents reported a negative complete cytogenetic response (CCyR), 280 (19.3%) respondents were in major molecular response (MMR), 810 (55.9%) respondents were in molecular response 4 (MR4). 213 (14.7%) respondents underwent TKI resistance. 241 (16.6%) respondents experienced TKI intolerance (Table 1). 544 respondents were treated with imatinib, 196 respondents were treated with dasatinib, 416 respondents received nilotinib, and 205 respondents received Flumatinib. 855 respondents were treated with full‐dose TKI. 542 respondents received low‐dose TKI according to ELN.^8^ 53 respondents were in TFR. 792 (54.6%) respondents reported a large to severe large financial burden of treatment (Table 2).

**TABLE 1:** Characteristics of the respondents.

	*N* = 1450
Male, *n* (%)	795 (54.8)
Age median, range (years)	44 (16–83)
Education, *n* (%)	
Junior middle school and below	505 (34.8)
Senior middle school	400 (27.6)
University and above	545 (37.6)
Living in rural area, *n* (%)	567 (39.1)
Marital status, *n* (%)	
Unmarried	220 (15.2)
Married	1168 (80.5)
Divorced or widowed	62 (4.3)
Disease phase at diagnosis, *n* (%)	
Chronic	1356 (93.5)
Accelerated	50 (3.4)
Blast	12 (0.8)
Unknown	32 (2.2)
TKI duration, mo, median (range)	50 (1–263)
Current TKI therapy line, *n* (%)	
First	810 (55.9)
Second	460 (31.7)
Third or Fourth	180 (12.4)
Response, *n* (%)	
<CCyR	89 (6.1)
≥CCyR, <MMR	219 (15.1)
≥MMR, <MR4	280 (19.3)
≥MR4	810 (55.9)
Unknown	52 (3.6)
History of TKI resistance, *n* (%)	213 (14.7)
History of TKI intolerance, *n* (%)	241 (16.6)

Abbreviations: 1G‐TKI, first‐generation TKI; 2G‐TKI, second‐generation TKI; 3G‐TKI, third‐generation TKI; CCyR, complete cytogenetic response; MMR, major molecular response; mo, month(s); MR4, molecular response 4; TKI, tyrosine kinase‐inhibitor.

**TABLE 2:** CML treatment.

Variables	*N*
Current medication	
Imatinib (Gleevec or generic imatinib)	544
100 mg/d	44 (8.1)
200 mg/d	30 (5.5)
300 mg/d	50 (9.2)
400 mg/d	400 (73.5)
500 mg/d	7 (1.3)
600 mg/d	13 (2.4)
Dasatinib (Sprycel or generic dasatinib)	196
20 mg/d	6 (3.1)
50 mg/d	85 (43.4)
70 mg/d	22 (11.2)
100 mg/d	78 (39.8)
140 mg/d	5 (2.5)
Nilotinib (Tasigna)	416
150 mg/d	45 (10.8)
200 mg/d	43 (10.3)
300 mg/d	63 (15.1)
400 mg/d	29 (7.0)
600 mg/d	172 (41.3)
800 mg/d	64 (15.4)
Flumatinib	205
200 mg/d	22 (10.7)
300 mg/d	4 (2.0)
400 mg/d	17 (8.3)
600 mg/d	162 (79.0)
Ponatinib	18
45 mg/d	13 (72.2)
30 mg/d	2 (11.1)
15 mg/d	3 (16.7)
Olverembatinib	
40 mg/bid	10
Others TKI	8
TKI discontinuation	53
The financial burden of treatment	
No	117 (8.1)
A little	541 (37.3)
Large	476 (32.8)
Severe large	316 (21.8)

*Note*: Olverembatinib is a third‐generation TKI in China.

### 3.2 History of TKI discontinuation and dose reduction

In this study, 124 patients underwent TKI discontinuation, 71 (57.3%) patients experienced relapse, and 53 (42.7%) patients remained in TFR. Patients discontinued TKI for reasons of relieving financial burden (35.5%), reducing adverse effects and improving QoL (51.6%), improving anxiety and depression while taking TKI (12.1%), worry about side effects of long‐term medication (41.9%), and pregnancy needs (9.7%). 583 patients experienced TKI dose reduction, 429 (73.6%) for reducing adverse effects and improving QoL, 344 (59.0%) for relieving financial stress, 377 (64.7%) for preparing for TKI discontinuation, and 109 (18.7%) patients for partially improving the inconvenience of TKI treatment. 28 patients returned to full‐dose treatment.

### 3.3 QoL and mental health in CML patients

We first analyzed the effect of TKI treatment on QoL, 154 (10.6%) respondents reported no impact, 653 (45.0%) reported a slight effect, 418 (28.8%) reported a moderate impact, 190 (13.1%) reported quite a bit impact, 35 (2.4%) reported severe impact. Fatigue, gastrointestinal reactions, edema, rash, and skeletal muscle soreness were frequent symptoms among CML patients (Table 3). Adverse events associated with imatinib, dasatinib, nilotinib, and flumatinib were shown in Table S1.

**TABLE 3:** QoL and self‐reported adverse events.

Variables	*N* = 1450
QoL, *n* (%)	
1	154 (10.6)
2	653 (45.0)
3	418 (28.8)
4	190 (13.1)
5	35 (2.4)
Adverse event, *n* (%)	
Fatigue	728 (50.2)
Edema	556 (38.3)
Gastrointestinal reactions	626 (43.2)
Rash	492 (33.9)
Skeletal muscle soreness	404 (27.9)
Leukopenia	147 (10.1)
Anemia	302 (20.8)
Thrombocytopenia	195 (13.4)
Abnormal liver function	266 (18.3)
Pleural effusion	111 (7.7)
Others	86 (5.9)

Abbreviation: QoL, quality‐of‐life.

When evaluating the mental health by PHQ‐9 and GAD‐7, 570 (39.3%) respondents reported no anxiety, 633 (43.7%) reported mild anxiety, 184 (12.7%) had moderate anxiety, and 63 (4.3%) had severe anxiety. 532 (36.7%), 565 (39.0%), 213 (14.7%), and 140 (9.7%) patients experienced normal, mild, moderate, and severe depression, respectively.

Then we evaluated the clinical factors potentially associated with poor QoL (score from 3 to 5) and moderate‐to‐severe depression. Univariate analysis results were shown in Table S2. In multivariate analyses, full‐dose TKI, history of TKI intolerance were significantly associated with poor QoL. Poor molecular response, full‐dose TKI, and the high financial burden were significantly associated with moderate‐to‐severe anxiety. Poor molecular response, full‐dose TKI, and the high financial burden were significantly associated with moderate‐to‐severe depression (Table 4).

**TABLE 4:** Factors associated with QoL, anxiety, and depression.

	Poor QoL		Anxiety		Depression	
	OR (95%)	*p*	OR (95%)	*p*	OR (95%)	*p*
Household registration (ref. Urban)				0.584		0.139
Rural			1.100 (0.782–1.549)		1.256 (0.929–1.697)	
Marital status (ref. Married)						0.172
Unmarried					0.928 (0.633–1.362)	0.704
Divorced or widowed					1.696 (0. 954–3.014)	0.072
Education (ref. Junior middle school and below)				0.928		0.528
Senior middle school			1.052 (0.720–1.538)	0.792	0. 919 (0.658–1.283)	0.618
University and above			0.978 (0.653–1.464)	0.914	0.811 (0.564–1.167)	0.260
Disease phase at diagnosis (ref. chronic)						
Advanced						
Blast						
Unknown						
TKI‐therapy duration (mo) (ref. ≤50)						
>50						
Current TKI (ref. 1G)						
2G						
3G and others						
TKI discontinuation						
Current TKI‐therapy line (ref. first)		0.138		0.037		0.954
Second	0.779 (0.608–0.997)	0.047	0.844 (0.591–1.205)	0.351	1.027 (0.756–1.393)	0.867
Third or fourth	0.902 (0.623–1.307)	0.587	1.553 (0.982–2.454)	0.060	0.961 (0.617–1.498)	0.861
Response (ref. <CCyR)				0. 006		<0.001
≥CCyR, <MMR			0. 478 (0.259–0.883)	0. 018	0.976 (0.549–1.732)	0.933
≥MMR, <MR4			0.336 (0.176–0.642)	0.001	0.793 (0.440–1.428)	0.440
≥MR4			0.341 (0.189–0.614)	<0.001	0.469 (0.269–0.818)	0.008
Unknown			0.296 (0.114–0.765)	0.012	0.602 (0.264–1.374)	0.228
TKI dose (ref. Full‐dose)		<0.001		<0.001		0.001
Low‐dose or TKI discontinuation	0.644 (0.513–0.807)		0.540 (0.392–0.743)		0.610 (0.458–0.813)	
TKI resistance (ref. No)		0.027		0.840		0.418
Yes	1.446 (1.044–2.003)		0.953 (0. 597–1.521)		1.187 (0.784–1.796)	
TKI intolerance (ref. No)		<0.001				0.076
Yes	1.753 (1.302–2.364)				1.387 (0.965–1.990)	
Financial burden (ref. Low)		0.013		<0.001		<0.001
High	1.31 (1.058–1.622)		7.228 (4.866–10.736)		4.704 (3.472–6.373)	

Abbreviations: 1G‐TKI, first‐generation TKI; 2G‐TKI, second‐generation TKI; 3G‐TKI, third‐generation TKI; CCyR, complete cytogenetic response; MMR, major molecular response; mo, month(s); MR4, molecular response 4; QoL, quality‐of‐life; TKI, tyrosine kinase‐inhibitor.

**FIGURE 1:**
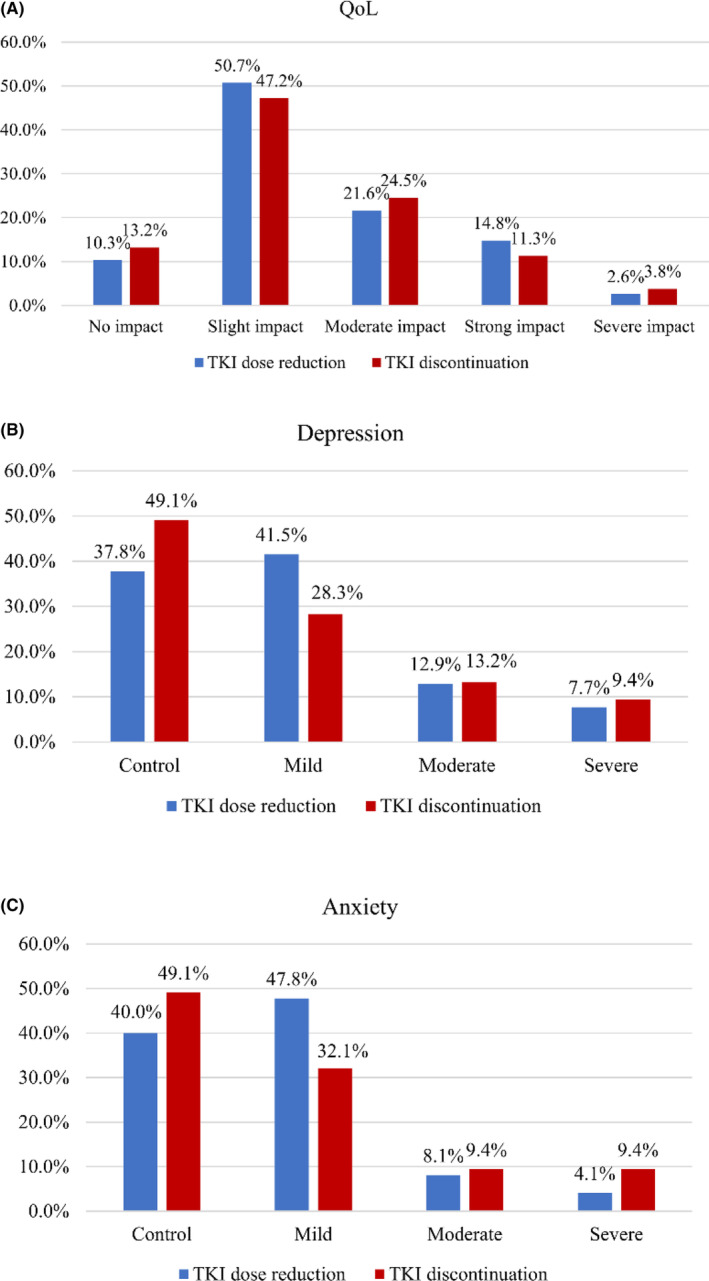
Comparison of QoL (A), depression (B), and anxiety (C) between respondents with TKI dose reduction and TKI discontinuation. QoL, quality‐of‐life; TKI, tyrosine kinase inhibitor.

In general, our results showed responders with low‐dose TKI or TFR experienced a low probability of poor QoL, moderate‐to‐severe anxiety, and depression compared to responders with full‐dose TKI. To analyze whether the QoL and mental health differed between patients with low‐dose TKI and TFR, we performed a subgroup analysis. The results showed that responders with dose reduction and TKI discontinuation have a similar probability of higher QoL, low to mild anxiety, and depression (Figure 1).

### 3.4 Treatment goals and patients' preference for TKI discontinuation and dose reduction

When asked what were the treatment goals: 61.7% (*n* = 895) of respondents reported TFR as their therapy‐goal, 70.4% (*n* = 1021) wished a normal life, 49.9% (*n* = 723) aimed at prevention of disease progression, and 26.7% (*n* = 378) had molecularly undetectable leukemia as treatment goals.

**FIGURE 2:**
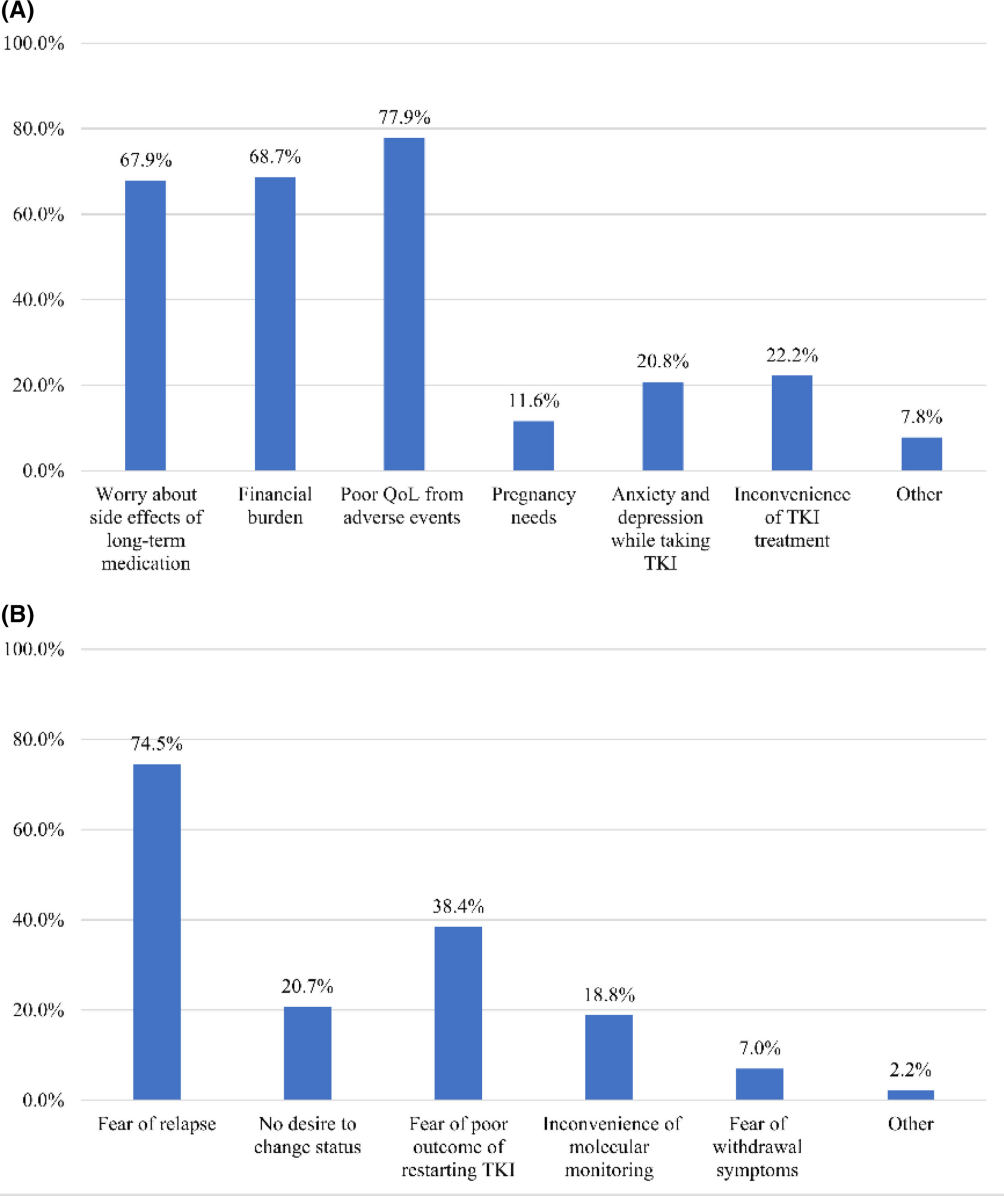
Reasons for respondents' preference to discontinue tyrosine kinase inhibitor therapy (A) or not (B). QoL, quality‐of‐life; TKI, tyrosine kinase inhibitor.

When asked the opinions on discontinuing TKI of the 1326 patients who had not previously discontinued TKI, 1055 (79.6%) patients reported they would try TKI discontinuation, for worry about side effects of long‐term medication (*n* = 716), financial burden (*n* = 725), poor QoL from adverse events (*n* = 822), pregnancy needs (*n* = 122), anxiety and depression while taking TKI (*n* = 219), inconvenience of TKI treatment (*n* = 234), and other (*n* = 82). Of the 122 patients who desired to discontinue TKI due to pregnancy needs, 45 patients were male and 67 were female. 271 patients were reluctant to discontinue the TKI due to fear of relapse (*n* = 202), no desire to change status (*n* = 56), fear of poor outcome of restarting TKI (*n* = 104), inconvenience of molecular monitoring (*n* = 51), and fear of withdrawal symptoms (*n* = 19) and other (*n* = 6) (Figure 2).

**FIGURE 3:**
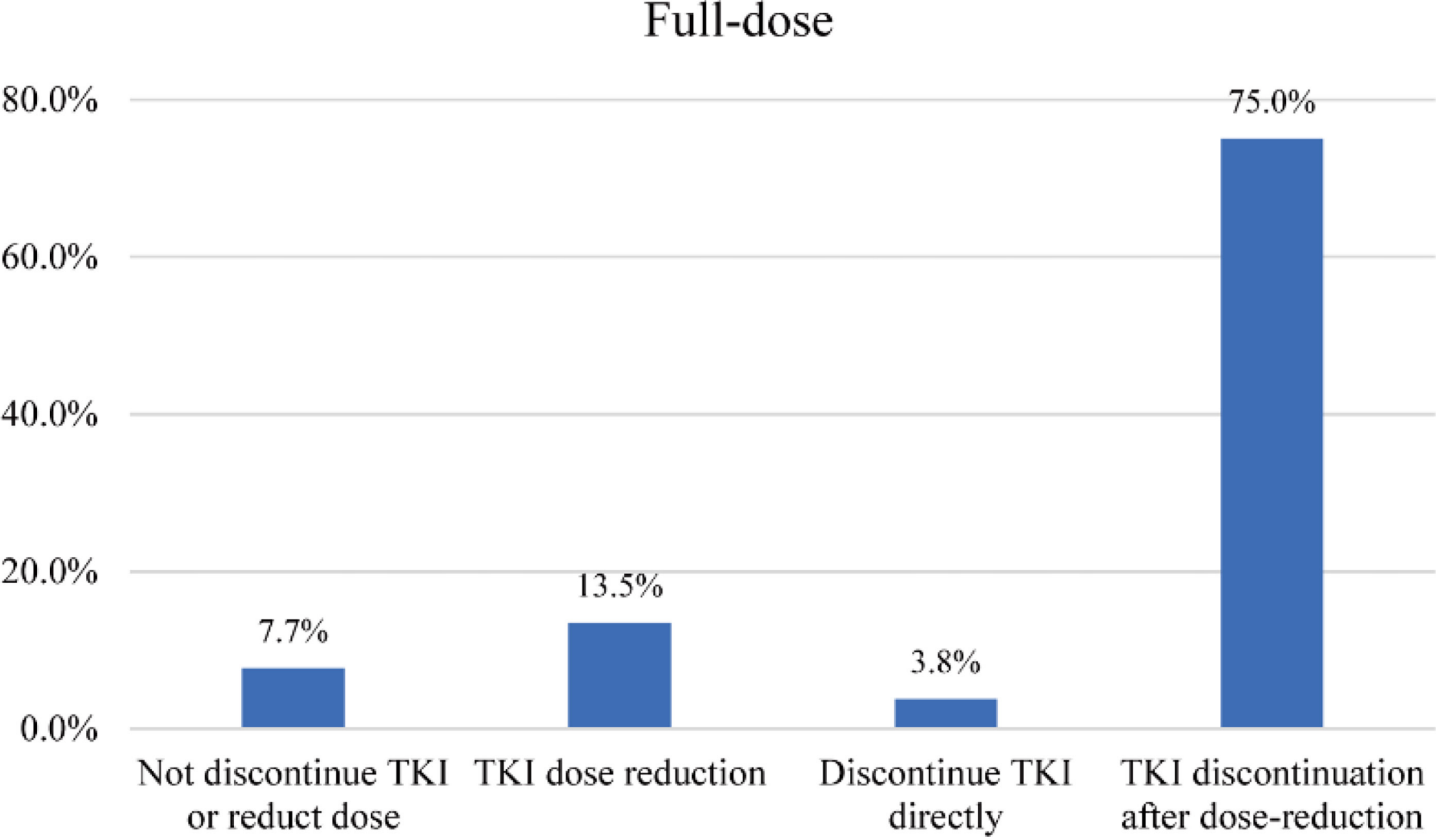
Preference for TKI discontinuation in patients with full‐dose TKI. TKI, tyrosine kinase inhibitor.

We further analyzed the patients' discontinuation models, as direct discontinuation or dose reduction followed by discontinuation. Overall, of the 1326 patients who did not discontinue the drug, 1055 (79.6%) patients were willing to discontinue TKI, of which 31 patients wanted to stop the drug directly. 271 (20.4%) did not wish to discontinue TKI, of which 208 preferred to reduce the dose of TKI, and 63 patients did not desire to either reduce or discontinue TKI. In 509 patients with low‐dose TKI, 411 patients preferred attempting TKI discontinuation, and 98 patients did not want to stop TKI. When 817 patients with full‐dose TKI were asked about their attitudes toward dose reduction or discontinuation, 63 patients did not desire to either reduce or discontinue TKI, 31 patients desired to stop TKI directly, 613 patients preferred to reduce the dose before discontinuing TKI, and 110 patients only wanted to reduce the dose without discontinuing the medication (Figure 3). Of the 71 patients who relapsed after stopping TKI, 58 still attempted to discontinue TKI in the future, including 56 patients who wanted to discontinue TKI after a dose reduction and 2 patients who wanted to stop TKI directly.

### 3.5 Factors associated with preference to discontinue TKI therapy in the future

Next, we analyzed the factors associated with the preference to discontinue TKI therapy in the future for patients who never experienced TKI discontinuation. In univariate and multivariate analyses, the factors associated with preference to discontinue TKI therapy in the future included unmarried status, long TKI therapy duration (>50 months), no history of TKI resistance, and poor QoL (Table 5).

**TABLE 5:** Factors associated with preference to discontinue TKI therapy in the future.

	TKI discontinuation		Multivariable analysis	
	*n* (%)	*p*	OR (95%)	*p*
Sex		0.480		
Male	586 (78.9)			
Female	469 (80.4)			
Age (years)		0.021		0.090
≤44 (ref.)	554 (82.1)		0.773 (0.574–1.041)	
>44	501 (77.0)			
Household registration		0.237		
Urban	632 (78.5)			
Rural	423 (81.2)			
Marital status		<0.001		
Married (ref.)	832 (78.0)			<0.001
Unmarried	185 (89.8)		2.523 (1.513–4.206)	<0.001
Divorced or widowed	38 (70.4)		0.566 (0.302–1.061)	0.076
Education		0.234		
Junior middle school and below	369 (79.2)			
Senior middle school	301 (82.5)			
University and above	385 (77.8)			
Disease phase at diagnosis of CML		0.471		
Chronic	979 (79.2)			
Advanced	41 (85.4)			
Blast	8 (72.7)			
Unknown	27 (87.1)			
TKI‐therapy duration (months)		<0.001		<0.001
≤50 (ref.)	515 (74.5)			
>50	540 (85.0)		2.114 (1.581–2.827)	
Current TKI		0.259		
1‐G	406 (78.7)			
2‐G	626 (80.6)			
3‐G and others	23 (69.7)			
TKI discontinuation	21 (39.6)			
Current TKI‐therapy line		0.993		
First	603 (79.7)			
Second	327 (79.4)			
Third or fourth	125 (79.6)			
Response		0.202		
<CCyR	55 (69.6)			
≥CCyR, <MMR	154 (78.2)			
≥MMR, <MR4	217 (81.3)			
≥MR4	591 (80.2)			
Unknown	38 (82.6)			
TKI dose		0.399		
Full‐dose	644 (78.8)			
Low‐dose	411 (80.7)			
TKI resistance		0.020		0.002
No (ref.)	915 (80.6)			
Yes	140 (73.3)		0.550 (0.379–0.798)	
TKI intolerance		0.613		
Yes	173 (80.8)			
No	888 (79.3)			
Financial burden		0.012		0.020
Low (ref.)	459 (76.5)			
High	596 (82.1)		1.397 (1.055–1.851)	
Poor QoL		<0.001		<0.001
No (ref.)	497 (74.1)		2.437 (1.810–3.280)	
Yes	558 (85.2)			
Moderate‐to‐severe anxiety		0.958		
Yes	180 (80.0)			
No	875 (79.5)			
Moderate‐to‐severe depression		0.799		
Yes	253 (79.1)			
No	802 (79.7)			

Abbreviations: 1G‐TKI, first‐generation TKI; 2G‐TKI, second‐generation TKI; 3G‐TKI, third‐generation TKI; CCyR, complete cytogenetic response; MMR, major molecular response; mo, month(s); MR4, molecular response 4; QoL, quality‐of‐life; TKI, tyrosine kinase‐inhibitor.

## 4 DISCUSSION

TFR has become the main target for CML as TKI therapy has dramatically improved survival rates for CML patients. In addition, evidence is accumulating that TKI dose optimization plays an important role in managing adverse events, and improving adherence.^22^ Importantly, it has been shown that dose reduction before TKI discontinuation does not impair the achievement of TFR.^17^, ^18^ However, few data are available on TKI discontinuation and dose reduction in Chinese CML patients. The current study has evaluated the characteristics of 1450 CML patients, medication status, QoL, adherence, and mental health and emphasized patient attitudes or preferences about TFR or TKI dose reduction in China. Most patients prefer to attempt to discontinue TKI in the future. Importantly, TKI discontinuation after dose reduction is more acceptable compared to discontinuing it directly.

Several real‐life TKI discontinuation studies have reported TFR rates of approximately 60%.^23^, ^24^ In this study, 124 patients underwent TKI discontinuation for different reasons, 71 (57.3%) patients experienced relapse, and 53 (42.7%) patients remained in TFR. In clinical practice, TKI dose adjustments are frequently associated with the alleviation of adverse events. Several studies in recent years have demonstrated that low‐dose TKI is effective in maintaining molecular responses. Naqvi et al. have reported an MMR rate of 81% and an MR4 rate of 55% in newly diagnosed CML‐CP patients treated with 50 mg dasatinib for 12 months.^25^ In the NILO‐RED study, the 12‐month survival rate without MMR loss among patients who switched from standard‐dose nilotinib to half‐dose nilotinib was 97%.^26^ Similarly, a real‐world retrospective study showed that 274 of 298 (91.9%) patients treated with low‐dose TKI maintained MMR at a median follow‐up of 27.3 months.^14^ In this study, of the 1450 respondents, 583 respondents underwent TKI dose reduction mainly for reducing adverse effects and improving QoL (73.6%), relieving financial stress (59.0%), preparing for TKI discontinuation (64.7%), improving the inconvenience of TKI treatment (18.7%). 28 (4.8%) patients lost MMR and returned to full‐dose treatment. Consistent with the literature our results showed that dose reduction was frequent in clinical practice and effective in maintaining response.

In contrast to previous studies that used patient‐reported outcome tools such as the EORTC QLQ‐C30 and EORTC QLQ‐CML24 to describe patients' QoL and symptom burden,^27^ here we used a simple score to grade patients' QoL and PHQ‐9, GAD‐7 scales to assess patients' mental health. We found that 49.2% of patients experienced poor QoL (score from 3 to 5). Fatigue, gastrointestinal reactions, edema, rash, and skeletal muscle soreness were frequent symptoms among CML patients. Similarly, fatigue was the most common symptom of patients with CML taking imatinib and second‐generation TKI.^28^ In a propensity‐matched case–control study assessing patients' QoL, dasatinib was found to have a significantly lower score for impact on daily life compared to imatinib.^29^ In a subgroup analysis of the ENESTnd trial, nilotinib 300 mg BID had a lower incidence for general disorders than imatinib.^30^ In this study, 2G‐TKI had a similar impact on QoL compared to imatinib. However, we found that full‐dose TKI, a history of TKI intolerance were significantly associated with poor QoL.

Anxiety and depression are two prevalent psychological disturbances in patients with CML. Phillips et al. reported that 37% of CML patients had self‐reported depression symptoms assessed using the CES‐D scale in America than noncancer participants.^31^ Another study reported that 22.4% of respondents experienced anxiety, including 3.8% with moderate‐to‐severe symptoms. 37.1% of respondents experienced depression, and 13.3% with moderate‐to‐severe depression on the SAS and the SDS scales.^32^ In this study, we used the PH7 and GAD‐9 scales to assess patients' anxiety and depression, respectively. We found that 60.7% of patients reported they had different levels of anxiety, and 17.0% were moderate‐to‐severe anxiety. 63.3% of respondents experienced different levels of depression, and 24.4% were moderate‐to‐severe depression. Similarly, poor molecular response, full TKI dose, and the high financial burden were significantly associated with moderate‐to‐severe anxiety. Poor molecular response, full TKI dose, and the high financial burden were significantly associated with moderate‐to‐severe depression. Not surprisingly, our results showed that TKI dose strongly affected QoL and mental health. Rare studies have explored the psychological state of patients who discontinue TKI. In a study comparing the anxiety‐depression status of patients discontinuing TKI using HADS scores, the HADS scores were significantly lower in patients with sustained TFR than those at the initiation of TKI discontinuation.^33^ Interestingly, we found responders with dose reduction and TKI discontinuation have a similar probability of higher QoL, low‐to‐mild anxiety, and depression. In general, we report for the first time the QoL and psychological status of patients with different treatment modalities. Compared to full‐dose treatment, discontinuing the drug improved patients' QoL and mental health, but did not show a significant advantage compared to dose reduction. However, a recent study reported that 56% of patients experienced fear or anxiety during TKI discontinuation. 59% felt scared or anxious, and 56% felt depressed while reinitiating treatment.^34^ Our results provide some evidence that dose reduction is also an alternative for patients who may be anxious or depressed during discontinuation.

When asked if 1326 patients who had not discontinued their medication would prefer to stop TKI in the future, most patients (79.6%) patients reported they would try TKI discontinuation, higher than in previous studies by our researchers. The reasons for TKI discontinuation mainly included worry about side effects of long‐term medication (67.9%), financial burden (68.7%), poor QoL from adverse reactions (77.9%), pregnancy needs (11.6%), anxiety and depression while taking the drug (20.8%), and improving the inconvenience of taking medication (22.2%). Similar to previous studies, unmarried, long TKI therapy duration, no history of TKI resistance, and poor QoL were significantly associated with preference to discontinue TKI therapy.^35^ Despite the increased availability of generic TKI with in China, long‐term treatment with TKI still increases the financial burden on patients and society. Currently, patients have to pay about 1000 RMB per month out of pocket for branded imatinib, 2000 RMB for branded second‐generation TKI, and 16,000 RMB for third‐generation TKI. For generic TKI, patients have to pay about 300 RMB per month out of pocket for imatinib and about 1000 RMB for second‐generation TKI. However, the average income of residents is about 2900 RMB a month, with about 3900 RMB for urban residents and 1500 RMB for rural residents. Undoubtedly, financial constraints are important incentives for patients to reduce TKI dose and discontinue TKI. From an economic point of view, an earlier reduction in TKI dose seems to provide more financial alleviation. However, the time point that patients can reduce their TKI dose remains inconclusive. A prospective study has shown that 50 mg of dasatinib daily is an effective and safe dose for newly diagnosed CML patients.^25^ Another prospective study, NILO‐RED, preliminary confirmed that a switch to nilotinib maintenance at a once daily dose is feasible and safe for patients who obtained MMR with standard doses of nilotinib.^26^ These limited prospective results demonstrated that low‐dose TKI is feasible and effective in newly diagnosed patients or patients with MMR. In our previous study, seven out of ten patients not in MMR achieved MMR or deeper molecular response with low‐dose treatment.^36^ In a study of patients with prior TKI intolerance treated with low‐dose ponatinib, 40.4% patients had improved treatment response over a median of 19.2 months of ponatinib treatment.^37^ These limited results suggested that low‐dose TKI could improve treatment response, but prospective studies with large samples are still needed to confirm. Overall, TKI dose reduction is safe, but the time to reduce dose still needs to be further explored in prospective studies. In clinical practice, TKI dose reduction also necessitates consideration of the patient's intention.

Recent evidence indicated that dose reduction before TKI discontinuation does not impair the achievement of TFR.^18^ Meanwhile, the majority of hematologists (64.4%) believed that low‐dose TKIs should not impact TFR.^38^ However, a rare study has explored patients' attitudes toward dose reduction and discontinuing the medication. In this study, of the 817 patients treated with full‐dose TKI, 63 patients did not desire to either reduce or discontinue the medication, 31 patients desired to stop TKI directly, 613 patients preferred to reduce the dose before discontinuing TKI, and 110 only wanted to reduce the dose without discontinuing the medication. Here we reported for the first time that TKI discontinuation after dose reduction is more acceptable to patients than direct discontinuation. Most patients who relapse after discontinuation can obtain DMR after reintroduction of TKI.^6^ Several studies showed that some patients who failed the first discontinuation could safely and successfully discontinue TKIs a second time.^39^, ^40^ In this study, 56/58 patients who relapsed after stopping TKI preferred second TKI discontinuation after a dose reduction. Importantly, 280 patients preferred to reduce the TKI dose rather than stop it, due to the fear of relapse after TKI discontinuation. In general, most patients considered TKI dose reduction and discontinuation after dose reduction to be safe and acceptable. In the updated DESTINY study, kinetic changes in BCR ABL1 IS values during dose reduction were strongly associated with relapse after TKI discontinuation.^41^ Changes in BCR‐ABL1 kinetics during dose reduction may help screen patients more suitable for discontinuation. In combination with the results in this study that patients favored TKI step‐down before discontinuation rather than direct discontinuation, TKI dose reduction can be considered as a bridge from full‐dose treatment to discontinuation. New indicators capable of screening for eligibility for TKI discontinuation during dose reduction still need to be addressed.

Although a large sample size was included in this study, some interesting results were obtained. The survey still has several limitations. Only 53 patients were in TFR, and the comparison of QoL and mental health between patients in TFR and patients with full or reduced doses may be biased. Hence a large sample of studies is still needed to confirm. In addition, the survey was conducted online only and introduced selection bias due to regional and socioeconomic factors. Respondents volunteered to participate in this study and therefore do not reflect the views of all CML patients.

Another limitation is the possible biased responses caused by respondent‐related factors. The accuracy of these responses may have important implications for the self‐reported questionnaire.

## AUTHOR CONTRIBUTIONS

**Yilin Chen:** Conceptualization (equal); data curation (equal); formal analysis (equal); methodology (equal); project administration (equal); writing – original draft (equal); writing – review and editing (equal). **Na Xu:** Conceptualization (equal); data curation (equal); writing – original draft (equal); writing – review and editing (equal). **Yunfan Yang:** Data curation (equal); formal analysis (equal); methodology (equal). **Zhenfang Liu:** Data curation (equal); formal analysis (equal). **Mengxing Xue:** Data curation (equal); formal analysis (equal). **Li Meng:** Data curation (equal); formal analysis (equal). **Qun He:** Data curation (equal); formal analysis (equal). **Chunyan Chen:** Data curation (equal); formal analysis (equal). **Qingshu Zeng:** Data curation (equal); formal analysis (equal). **Huanling Zhu:** Data curation (equal); formal analysis (equal). **Xin Du:** Data curation (equal); formal analysis (equal). **Jing Zou:** Data curation (equal); formal analysis (equal). **Wenjun He:** Data curation (equal); formal analysis (equal). **Jingming Guo:** Data curation (equal); formal analysis (equal). **Suning Chen:** Data curation (equal); formal analysis (equal). **Guolin Yuan:** Data curation (equal); formal analysis (equal). **Hui Wu:** Data curation (equal); formal analysis (equal). **Mei Hong:** Data curation (equal); formal analysis (equal). **Fanjun Cheng:** Conceptualization (equal); data curation (equal); formal analysis (equal); methodology (equal); writing – original draft (equal); writing – review and editing (equal). **Bingcheng Liu:** Conceptualization (equal); data curation (equal); formal analysis (equal); writing – original draft (equal); writing – review and editing (equal). **Yanli Zhang:** Conceptualization (equal); data curation (equal); formal analysis (equal); writing – original draft (equal); writing – review and editing (equal). **Weiming Li:** Conceptualization (equal); data curation (equal); formal analysis (equal); writing – original draft (equal); writing – review and editing (equal).

## FUNDING INFORMATION

This work was supported by the National Key Research and Development Plan of China (Grant number. 2020YFC2006000).

## CONFLICT OF INTEREST STATEMENT

The authors declare that they have no conflict of interest.

## Supporting information


Data S1.

## Data Availability

The data used to support the findings of this study are available from the corresponding author upon reasonable request.
