# Supplementary material for: Quality‐of‐life, mental health, and perspective on TKI dose reduction as a prelude to discontinuation in chronic phase chronic myeloid leukemia
Source: Cancer Med. 2023 Jul 6;12(16):17239–52. doi: 10.1002/cam4.6296 (PMC10501272; doi:10.1002/cam4.6296)
Supplement: Supplementary file 1 — Data S1. [file CAM4-12-17239-s001.docx]

Supplementary Material

# Supplementary Material

**1.1**

**Questionnaire on patients’ quality of life, mental health and intentions to dose reduction and discontinuation of TKI**

**Part 1**

With the introduction of tyrosine kinase inhibitors (TKIs), chronic myeloid leukemia (CML) has become a manageable chronic disease. Most patients can achieve long-term survival with standard therapy. It has been shown that about half of patients who obtain a deep treatment response can successfully discontinue the drug. Relapse after discontinuation and a high threshold for discontinuation are also important constraints to discontinuation. In recent years, dose reduction therapy has also been increasingly emphasized, and most patients can maintain efficacy with dose reduction therapy. However, limited date on TKI discontinuation and dose reduction is available in China based on patient’s perspective. We conducted a cross-sectional study to explore the quality of life and mental health of Chinese patients with CML, as well as patient perceptions of dose reduction and discontinuation of TKI.

**Part 2**

**Patients’ and treatment characteristics**

1. Gender?

Male

Female

1. Age? _____________________________
2. Education

Junior middle school and below

Senior middle school

University and above

1. Household registration?

Urban

Rural

1. Marital status?

Married

Unmarried

Divorced or widowhood

1. Disease phase at diagnosis of CML

Chronic

Accelerated

Blast

Unknown

7.When did you start the TKI-therapy (imatinib, dasatinib, nilotinib, or others)?

__________ Year ___________Month

8.Which TKI drug are you taking now?

Glivec ___________mg

Xinwei (Chinese generic imatinib) ___________mg

GeNiKe (Chinese generic imatinib) ___________mg

Nuolining (Chinese generic imatinib) ___________mg

Foreign generic imatinib___________mg

Sprycel ___________mg

Yinishu (Chinese generic dasatinib) ___________mg

Foreign generic dasatinb___________mg

Tasgina ___________mg

Ponatinib ___________mg

Flumatinib___________mg

HQP1351 ___________mg

Discontinuation of TKI-therapy

___________

9.Current TKI therapy line, n (%)

1st

2nd

3rd or 4th

10. Have a history of TKI resistance

Yes

No

11. Have a history of TKI intolerance

Yes

No

12. The last testing results of BCR-ABL_1_ _____% (IS)

13. How about the financial burden of treatment (1=no, 4= severe large)

1

2

3

4

**History of TKI discontinuation and TKI dose reduction**

1. Have you experienced TKI discontinuation?

Yes

No

1. What were reasons for TKI discontinuation? (Please select the top three)

To relieve financial burden

To reduce adverse effects and improve quality of life

Pregnancy needs

To improve the inconvenience of taking TKI

To improve anxiety and depression of taking TKI

Fear of side effects of long-term medication

_____________

1. Did you experience a relapse (loss of MMR) after stopping TKI and resuming TKI?

Yes

No

1. Have you experienced TKI dose-reduction?

Yes

No

1. What were reasons for TKI dose-reduction? (Please select the top three)

To relieve financial burden

To reduce adverse effects and improve quality of life

Pregnancy needs

To improve partially the inconvenience of taking TKI

_____________

1. Did you experience a relapse (loss of MMR) after TKI dose-reduction and returning to full-dose?

Yes

No

**Patient’s self-reported events such as QoL and mental health**

20. Your major drug adverse reactions include: (Please select the top three)

Fatigue

Edema

Gastrointestinal reactions

Rash

Skeletal muscle soreness

Leukopenia

Anemia

Thrombocytopenia

Hepatic abnormality

Pleural effusion

_____________

21.Please grade the impact of AE on quality of life (1 = no impact; 5 = high impact):

1

2

3

4

5

1. **7-item Generalized Anxiety Disorder (GAD-7)**

Feeling nervous, anxious, or on edge

Not at all—0

Several days—1

More than half the days—2

Nearly every day—3

Inability to stop or control worrying

Not at all—0

Several days—1

More than half the days—2

Nearly every day—3

Worrying too much about different things

Not at all—0

Several days—1

More than half the days—2

Nearly every day—3

Trouble relaxing

Not at all—0

Several days—1

More than half the days—2

Nearly every day—3

Being so restless that it is hard to sit still

Not at all—0

Several days—1

More than half the days—2

Nearly every day—3

Becoming easily annoyed or irritable

Not at all—0

Several days—1

More than half the days—2

Nearly every day—3

Feeling afraid as if something awful might happen

Not at all—0

Several days—1

More than half the days—2

Nearly every day—3

1. **9-item Patient Health Questionnaire (****PHQ-9)**

Little interest or pleasure in doing things

Not at all—0

Several days—1

More than half the days—2

Nearly every day—3

Feeling down, depressed, or hopeless

Not at all—0

Several days—1

More than half the days—2

Nearly every day—3

Trouble falling or staying sleeping too much

Not at all—0

Several days—1

More than half the days—2

Nearly every day—3

Feeling tired or having little energy

Not at all—0

Several days—1

More than half the days—2

Nearly every day—3

Poor appetite or overeating

Not at all—0

Several days—1

More than half the days—2

Nearly every day—3

Feeling bad about yourself or that you are a failure or have let yourself or your family down

Not at all—0

Several days—1

More than half the days—2

Nearly every day—3

Trouble concentrating on things, such as reading the newspaper or watching television

Not at all—0

Several days—1

More than half the days—2

Nearly every day—3

Moving or speaking so slowly that other people could have noticed? Or the opposite being so fidgety or restless that you have been moving around a lot more than usual

Not at all—0

Several days—1

More than half the days—2

Nearly every day—3

Thoughts that you would be better off dead, or of hurting yourself in some way

Not at all—0

Several days—1

More than half the days—2

Nearly every day—3

**Preference of TFR and dose reduction and reason**

1. What is your goal for CML treatment? (Please select the top three)

To prevent disease progression

Negative BCR-ABL gene detection (i.e. complete molecular response)

To achieve a normal life span

To discontinue TKI therapy with disease remission

1. Do you hope to stop TKI in the future?

Yes

No

1. If you want to stop TKI, why? (Please select the top three)

warry about side effects of long-term medication

Financial burden

Poor quality of life from adverse reactions

Planned needs

Anxiety and depression of TKI treatment

Inconvenience of TKI treatment

_____________

1. If you want to stop TKI, which of the following would you prefer?

Directly discontinue TKI

TKI discontinuation after dose reduction

1. If you do not want to stop TKI, what is your major concern? (Please select the top three)

Fear of relapse

No desire to change status

Fear of poor outcome of restarting TKI

inconvenience of molecular monitoring

fear of withdrawal symptoms

_____________

1. If you do not want to stop TKI, are you willing to reduce TKI dose?

Yes

No

**Supplementary Table 1 TKI-related adverse events**

| Adverse event n (%) | Imatinib | Dasatinib | Nilotinib | Flumatinib |
| --- | --- | --- | --- | --- |
| Fatigue | 276(50.7) | 103(52.6) | 203(48.8) | 101(49.3) |
| Edema | 372(68.4) | 82(41.8) | 38(9.1) | 35(17.1) |
| Gastrointestinal reactions | 281(51.7) | 81(41.3) | 105(25.2) | 129（62.9） |
| Rash | 234(43.0) | 66(33.7) | 109(26.2) | 54(26.3) |
| Skeletal muscle soreness | 150(27.6) | 58(29.6) | 108(26.0) | 62(30.2) |
| Leukopenia | 83(15.3) | 20(10.2) | 17(4.1) | 16(7.8) |
| Anemia | 141(25.9) | 46(23.5) | 55(13.2) | 38(18.5) |
| Thrombocytopenia | 46(8.5) | 40(20.4) | 49(11.8) | 37(18.1) |
| [Abnormal liver function](http://www.youdao.com/w/abnormal%20liver%20function/#keyfrom=E2Ctranslation) | 35(6.4) | 25(12.8) | 171(41.1) | 19(9.3) |
| Pleural effusion | 33(6.1) | 45(23.0) | 23(5.5) | 10(4.9) |
| Others | 30(5.5) | 15(7.7) | 22(5.3) | 11(5.4) |

TKI= tyrosine kinase inhibitors.

**Supplementary Table 2.** Univariate analyses of QoL and mental health

|  | Poor QoL | | Anxiety | | | | Depression | | | |  |
| --- | --- | --- | --- | --- | --- | --- | --- | --- | --- | --- | --- |
|  | n (%) *P* | | n (%) *P* | | | | n (%) *P* | | | |  |
| Sex |  | 0.487 |  | | 0.521 | |  | | 0.195 | |  |
| Male | 346 (43.5) |  | 140 (17.6) | |  | | 183 (23.0) | |  | |  |
| Female | 297 (45.3) |  | 107 (16.3) | |  | | 170 (26.0) | |  | |  |
| Age(years) |  | 0.075 |  | | 0.133 | |  | | 0.227 | |  |
| ≤44 | 304 (42.0) |  | 134 (18.5) | |  | | 186 (25.7) | |  | |  |
| ＞44 | 339 (46.7) |  | 113 (15.6) | |  | | 167 (23.0) | |  | |  |
| Household registration |  | 0.621 |  | | 0.002 | |  | | 0.000 | |  |
| Urban | 387 (43.8) |  | 129 (14.6) | |  | | 178 (20.2) | |  | |  |
| Rural | 256 (45.1) |  | 118 (20.8) | |  | | 175 (30.9) | |  | |  |
| Marital status |  | 0.080 |  | | 0.197 | |  | | 0.038 | |  |
| Married | 525 (44.9) |  | 203 (17.4) | |  | | 283 (24.2) | |  | |  |
| Unmarried | 85 (38.6) |  | 30 (13.6) | |  | | 47 (21.4) | |  | |  |
| Divorced or widowed | 33 (53.2) |  | 14 (22.6) | |  | | 23 (37.1) | |  | |  |
| Education |  | 0.088 |  | | 0.032 | |  | | 0.000 | |  |
| Junior middle school and below | 216 (42.8) |  | 101(20.0) | |  | | 154 (30.5) | |  | |  |
| Senior middle school | 196 (49.0) |  | 70 (17.5) | |  | | 98 (24.5) | |  | |  |
| University and above | 231 (42.4) |  | 76 (13.9) | |  | | 101 (18.5) | |  | |  |
| Disease phase at diagnosis |  | 0.951 |  | | 0.545 | |  | | 0.622 | |  |
| Chronic | 604 (44.5) |  | 236 (17.4) | |  | | 334 (24.6) | |  | |  |
| Advanced | 21(42.0) |  | 6 (12.0) | |  | | 12 (24.0) | |  | |  |
| Blast | 5 (41.7) |  | 1 (8.3) | |  | | 2 (16.7) | |  | |  |
| Unknown | 13 (40.6) |  | 4 (12.5) | |  | | 5 (15.6) | |  | |  |
| TKI-therapy duration (mo) |  | 0.220 |  | | 0.117 | |  | | 0.591 | |  |
| ≤50 | 326 (46.0) |  | 132(18.6) | |  | | 177 (25) | |  | |  |
| ＞50 | 317 (42.8) |  | 115(15.5) | |  | | 176 (23.8) | |  | |  |
| Current TKI |  | 0.104 |  | | 0.067 | |  | | 0.229 | |  |
| 1G | 263 (48.3) |  | 89(16.4) | |  | | 132 (24.3) | |  | |  |
| 2G | 342 (41.9) |  | 136 (16.6) | |  | | 195 (23.9) | |  | |  |
| 3G and others | 17 (47.2) |  | 12 (33.3) | |  | | 14 (38.9) | |  | |  |
| TKI discontinuation | 21 (39.6) |  | 10 (18.9) | |  | | 12 (22.6) | |  | |  |
| Current TKI-therapy line |  | 0.043 |  | | 0.000 | |  | | 0.047 | |  |
| 1st | 367 (45.3) |  | 131 (16.2) | |  | | 182 (22.5) | |  | |  |
| 2nd | 185 (40.2) |  | 67 (14.6) | |  | | 115 (25) | |  | |  |
| 3rd or 4th | 91 (50.6) |  | 49 (27.2) | |  | | 56 (31.1) | |  | |  |
| Response |  | 0.452 |  | | 0.000 | |  | | 0.000 | |  |
| <CCyR | 43 (48.3) |  | 29 (32.6) | |  | | 32 (36.0) | |  | |  |
| ≥CCyR, <MMR | 92 (42.0) |  | 49 (22.4) | |  | | 77 (35.2) | |  | |  |
| ≥MMR, <MR4 | 135 (48.2) |  | 45 (16.1) | |  | | 82 (29.3) | |  | |  |
| ≥MR4 | 353 (43.6) |  | 116 (14.3) | |  | | 148 (18.3) | |  | |  |
| unknow | 20 (38.5) |  | 8 (15.4) | |  | | 14 (26.9) | |  | |  |
| TKI dose |  | 0.001 |  | | <0.001 | |  | | 0.009 | |  |
| Full-dose | 411(48.1) |  | 171 (20.0) | |  | | 229 (26.8) | |  | |  |
| Low-dose or TKI discontinuation | 232 (39.0) |  | 76 (12.8) | |  | | 124 (20.8) | |  | |  |
| TKI resistance |  | 0.013 |  | | 0.000 | |  | | 0.000 | |  |
| Yes | 111 (52.1) |  | 54 (25.4) | |  | | 74 (34.7) | |  | |  |
| No | 532 (43.0) |  | 193(15.6) | |  | | 279 (22.6) | |  | |  |
| TKI intolerance |  | 0.004 | |  | | 0.093 | |  | | 0.028 | |
| Yes | 127 (52.7) |  | 50 (20.7) | |  | | 72 (29.9) | |  | |  |
| No | 516 (42.7) |  | 197(16.3) | |  | | 281(23.2) | |  | |  |
| Financial burden |  | 0.012 |  | | 0.000 | |  | | 0.000 | |  |
| Low | 268 (40.7) |  | 33 (5.0) | |  | | 66 (10.0) | |  | |  |
| High | 375 (47.3) |  | 214 (27.0) | |  | | 287(36.2) | |  | |  |

*QOL, quality of life; mo, month(s); TKI, tyrosine kinase-inhibitor; 1G-TKI, firs-generation TKI; 2G-TKI, second-generation TKI; 3G-TKI, third-generation TKI; CCyR, complete cytogenetic response; MMR, major molecular response; MR4, molecular response 4.
